# Biodegradable polymer drug-eluting stents versus second-generation drug-eluting stents in patients with and without diabetes mellitus: a single-center study

**DOI:** 10.1186/s12933-018-0758-0

**Published:** 2018-08-14

**Authors:** Xiao-Fang Tang, Yuan-Liang Ma, Ying Song, Jing-Jing Xu, Yi Yao, Chen He, Huan-Huan Wang, Ping Jiang, Lin Jiang, Ru Liu, Zhan Gao, Xue-yan Zhao, Shu-Bin Qiao, Yue-Jin Yang, Run-Lin Gao, Bo Xu, Jin-Qing Yuan

**Affiliations:** 1 State Key Laboratory of Cardiovascular Disease, Department of Cardiology, Centre for Coronary Heart Disease, Fuwai Hospital, National Center for Cardiovascular Diseases, Chinese Academy of Medical Sciences and Peking Union Medical College, Beilishi Road 167, Xicheng District, Beijing, Postal code: 100037 China; 2 Department of Cardiology, Xuanwu Hospital Capital Medical University, No. 45 Changchun Road, Xicheng District, Beijing, China

**Keywords:** Diabetes mellitus, Second-generation drug-eluting stent, Biodegradable polymer drug-eluting stent, Target lesion revascularization

## Abstract

**Background:**

To improve outcomes in patients with diabetes mellitus (DM) undergoing percutaneous coronary intervention remain an unmet clinical need. The study aimed to evaluate the efficacy and safety of G2-DESs and BP-DESs in patients with and without DM in a single center in China.

**Methods:**

A total of 7666 consecutive patients who exclusively had G2-DES or BP-DES implantation throughout 2013 in our center were studied. The primary efficacy endpoint was any target lesion revascularization (TLR), whereas the primary safety endpoint was a composite of death or myocardial infarction (MI) at 2-year follow-up.

**Results:**

G2-DESs had a similar occurrence of death, non-fatal MI, TLR, stroke, and stent thrombosis compared with BP-DESs in patients with DM (all P > 0.05). The incidence of TVR and TLR was lower for G2-DESs than for BP-DESs in patients without DM (3.2% vs. 5.1%, P = 0.002; 2.2% vs. 4.5%, P < 0.001, respectively). Kaplan–Meier analysis also showed better TVR- and TLR-free survival rates for G2-DESs than for BP-DESs in patients without DM. Multivariate analysis showed that a BP-DES was an independent risk factor for TLR (hazard ratio 1.963, 95% confidence interval 1.390–2.772, P < 0.001) in patients without DM, which was not predictive of other components of major adverse cardiac events (P > 0.05).

**Conclusions:**

G2-DESs have better efficacy, represented by a reduced risk of TLR, and similar safety compared with BP-DESs in patients without DM. G2-DESs have similar efficacy and safety compared with BP-DESs in patients with DM at 2-year follow-up.

**Electronic supplementary material:**

The online version of this article (10.1186/s12933-018-0758-0) contains supplementary material, which is available to authorized users.

## Background

Coronary stents have been used for more than two decades. During this period, stent designs have been modified to improve safety in patients [1]. Bare metal stents (BMSs) were followed by first-generation permanent polymer drug-eluting stents (paclitaxel- and sirolimus-DESs). These stents were then followed by second-generation permanent polymer DESs (everolimus- and zotarolimus-DESs). Currently, biodegradable polymer DESs (BP-DESs) are being used and potentially improve patients’ outcomes.

First-generation DESs decrease the risk of stent restenosis and ischemia-driven target lesion revascularization (TLR) compared with BMSs in a broad range of patients and coronary lesions [2]. Second-generation DESs (G2-DESs), such as everolimus- and zotarolimus-eluting stents, markedly decrease the risk of early stent thrombosis and repeat revascularization compared with first-generation DESs and BMSs [3–6]. Nevertheless, these new stents still have limitations, not least the requirement for prolonged dual-antiplatelet therapy and an apparent increase in the incidence of Academic Research Consortium definite early and late stent thrombosis [7]. These problems are likely caused by delayed vessel healing, impaired endothelialization, allergy, inflammation, and the presence of durable polymers [8, 9]. DESs using biodegradable polymers were designed to overcome long-term adverse vascular reactions when drug elution is complete. These stents may reduce the risk of in-stent restenosis and be non-inferior to in-segment late loss after deployment [10–13].

Interventional treatment of patients with coronary artery disease and diabetes mellitus (DM) remains a challenge. This group of patients suffers from a greater burden and more rapid progression of coronary atherosclerosis compared with non-diabetic patents. DM is associated with an increased risk of in-stent restenosis, TLR, and target vessel revascularization (TVR) in patients undergoing percutaneous coronary intervention (PCI) [14–16]. Therefore, adverse outcomes after coronary revascularization in patients with DM remain a concern regarding which type of DES to use [17]. Whether BP-DESs are potentially superior to G2-DESs still remains undetermined in patients with DM. Therefore, this study aimed to compare long-term safety and efficacy after PCI with G2-DESs and BP-DESs in an unrestricted, real-life, single-center population with or without DM in China.

## Methods

### Study population

**Fig. 1:**
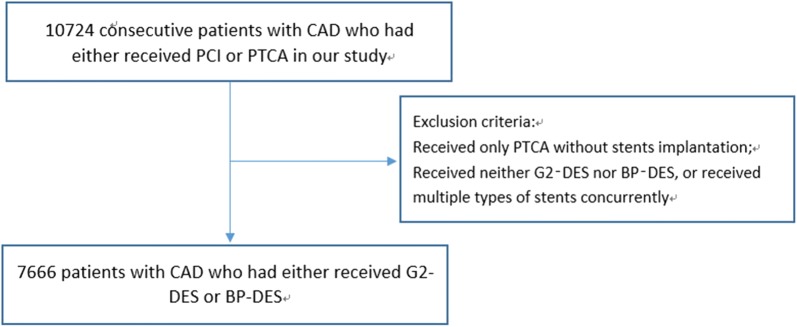
Flowchart of the study. *CAD* coronary artery disease, *PCI* percutaneous coronary intervention, *PTCA* percutaneous transluminal coronary angioplasty, *G2*-*DES* second-generation drug-eluting stent, *BP*-*DES* biodegradable polymer drug-eluting stent

This was a prospective, observational study. Data from all consecutive patients from a single center who underwent PCI were prospectively collected. Between January 1, 2013, and December 31, 2013, a total of 10,724 consecutive patients who underwent PCI were enrolled. Exclusion criteria included the following: (1) patients who received only percutaneous transluminal coronary angioplasty without stent implantation; and (2) patients who received neither G2-DESs nor BP-DESs, or received multiple types of stents concurrently. A total of 7666 patients were enrolled who received either G2-DESs (n = 6094) or BP‑DESs (n = 1572) (Fig. 1). DM was diagnosed on the basis on previous medical records of the patients and data of the therapeutic status based on the drug regimen of glucose-lowering therapy (including insulin, oral antibiotics, and diet and exercise). The stent type and operating strategy were left to the operators’ discretion. In our center, G2-DESs included zotarolimus-eluting stents (Endeavor and Endeavor Resolute; Medtronic Vascular, USA), everolimus-eluting stents (Xience V and Xience Prime; Abbott Vascular, USA, and Promus and Promus Element; Boston Scientific, USA), and domestic sirolimus-eluting stents (Firebird2; MicroPort Medical, China). BP-DESs included sirolimus-eluting stents (FIREHAWK; MicroPort Medical, China, Excel; JW Medical, China, BuMA; Sino Medical, China, NOYA; Medfavour Medical, China, and Tivoli; Essen Technology, China). If patients received PCI treatment in multiple stages because of multivessel disease, we combined the data from all phases of treatment. The study protocol was approved by the institutional review board of our hospital, and all patients provided written informed consent before the study.

### Procedural details

The PCI strategy and stent type were left to the discretion of the operating surgeon. Before the procedure, selected patients with PCI who were not on long-term aspirin and/or P2Y12 inhibitors received oral administration of aspirin 300 mg and clopidogrel (loading dose of 300 mg). Patients with acute coronary syndrome (ST-elevation myocardial infarction and NSTE-acute coronary syndrome) who were scheduled for PCI received the same dose of aspirin and clopidogrel (loading dose of 300 or 600 mg) as soon as possible. During the procedure, unfractionated heparin (100 U/kg) was administered to all of the patients, and glycoprotein IIb/IIIa inhibitors were used according to the operator’s judgment. If PCI proceeded for longer than 1 h, an additional 1000 U of heparin sodium was administered. Results of coronary angiography were interpreted by experienced cardiologists. More than 50% stenosis of the left main artery, left anterior descending artery, left circumflex artery, right coronary artery, and main branch of these vessels was defined as coronary artery stenosis. More than 70% stenosis of these vessels was indicated for coronary stent implantation. After the procedure, aspirin was prescribed at a dose of 100 mg daily indefinitely, and clopidogrel 75 mg daily or ticagrelor 90 mg twice daily for at least 1 year after PCI was recommended.

### Follow-up and definitions

All of the patients were evaluated by a visit to the clinic or by phone 1, 3, 6, and 12 months postoperatively and annually thereafter up to 2 years. Follow-up data were collected through medical records, telephone calls, or clinical visits, and an independent group of follow-up investigators were in charge of data collection and revision. Patients were advised to return for coronary angiography if indications of ischemic events occurred. The composite of major adverse cardiac events (MACE) was defined as the occurrence of death, nonfatal myocardial infarction (MI), TLR, stent thrombosis, and stroke during follow-up (730 ± 30 days). MI was defined by the Third Universal Definition of myocardial infarction [18]. TVR was defined as revascularization for a new lesion on the target vessel either by PCI or by coronary artery bypass grafting (CABG). TLR was defined as revascularization for a new lesion at or within 5 mm from the previously implanted stent either by PCI or by CABG [19]. Stent thrombosis included definite, probable, and possible stent thrombosis based on the Academic Research Consortium criteria [19]. Death that could not be attributed to a noncardiac etiology was considered cardiac death. Bleeding was quantified according to Bleeding Academic Research Consortium (BARC) criteria [20]. In the present study, major bleeding was defined as BARC type 2, 3, or 5 (type 4, CABG-related bleeding, was excluded). The efficacy endpoint was TLR and the safety endpoint was a composite of death or MI at 2 years. Two independent physicians who were blinded to the laboratory data adjudicated events after reviewing the source documents.

### Statistical analysis

Continuous variables are presented as mean ± standard deviation and were compared using the Student’s t-test or one-way analysis of variance, as appropriate. Categorical variables are expressed as numbers and percentages and were compared using the Chi square test or Fisher’s exact test. After significant differences among variables were shown using one-way analysis of variance, post hoc comparisons between groups were performed using the Student–Newman–Keuls method for multiple comparisons. The event-free survival rates were calculated by the Kaplan–Meier method and were compared by the log-rank test. The Cox proportional regression model was used to assess the independent predictors of endpoint events. Variables that had P values of < 0.10 in univariate analysis were included in multivariate COX regression analysis based on the backward stepwise method. All P values were two-sided and P < 0.05 was considered statistically significant. All statistical analyses were performed using SPSS Statistics for Windows, Version 22.0 (IBM Corp., Armonk, NY, USA).

## Results

### Baseline, angiographic and procedural characteristics of the patients

Among a total of 7666 patients, 2283 (29.8%) patients had DM. A total of 1862 patients with DM used G2-DESs. Among these patients, the Endeavor was used in 4.5%, the Endeavor Resolute in 31.3%, the Xience V in 23.2%, the Xience Prime in 9.9%, the Promus in 0.1%, the Promus Element in 8.8%, and the Firebird2 in 22.2%. A total of 421 patients with DM used BP-DESs. Among these patients, the Excel was used in 75.8%, the BuMA in 5.2%, the NOYA in 1.9%, and the Tivoli in 17.1%. A total of 4232 patients without DM used G2-DESs. Among these patients, the Endeavor was used in 3.6%, the Endeavor Resolute in 30.9%, the Xience V in 22.2%, the Xience Prime in 11.4%, the Promus Element in 9.4%, and the Firebird2 in 22.5%. A total of 1151 patients without DM used BP-DESs. Among these patients, the Excel was used in 69.1%, the BuMA in 8.1%, the NOYA in 1.9%, and the Tivoli in 20.9%.

With regard to baseline characteristics in patients with DM, the BP-DES group had a significantly higher proportion of hypertension (P = 0.023), a history of old myocardial infarction (OMI) (P = 0.013), and previous PCI (P = 0.015) than did the G2-DES group. In patients without DM, the BP-DES group had a significantly higher proportion of ST-elevation myocardial infarction (P = 0.03) and a lower proportion of a history of previous CABG (P = 0.006) than did the G2-DES group. With regard to medication, the proportions of using β‑blockers (P = 0.004) and GP IIb/IIIa inhibitors (P = 0.035) were significantly higher in the BP-DES group than in the G2-DES group in patients with DM. The proportions of using dual-antiplatelet therapy (P = 0.018), GP IIb/IIIa inhibitors (P < 0.001), and proton pump inhibitors (P = 0.012) were significantly higher in the BP-DES group than in the G2-DES group in patients without DM (Table 1).

**Table 1:** Clinical baseline characteristic and medication data

Characteristics	DM		*P*	No-DM		*P*
	G2‑DES N = 1862	BP‑DES N = 421		G2‑DES N = 4232	BP‑DES N = 1151	
Age (years)	59.0 ± 9.7	59.7 ± 9.7	0.814	57.5 ± 10.6	58.5 ± 10.4	0.241
Sex (male)	1411 (75.8)	314 (74.6)	0.607	3307 (78.1)	872 (75.8)	0.085
BMI (kg/m^2^)	26.3 ± 3.1	26.2 ± 3.1	0.595	25.8 ± 3.2	25.8 ± 3.1	0.754
LVEF (%)	62.7 ± 7.5	61.8 ± 7.9	0.207	63.1 ± 7.2	61.8 ± 7.9	0.262
Hypertension (n, %)	1284 (69.0)	314 (74.6)	0.023	2545 (60.1)	726 (63.1)	0.070
Hyperlipidemia (n, %)	1383 (74.3)	306 (72.7)	0.502	2754 (65.1)	717 (62.3)	0.080
Current smoker (n, %)	1014 (54.5)	240 (57.0)	0.342	2414 (57.0)	685 (59.5)	0.132
Family history (n, %)	486 (26.1)	102 (24.2)	0.427	1047 (24.7)	264 (22.9)	0.206
Stroke history (n, %)	231 (12.4)	60 (14.3)	0.305	369 (8.7)	118 (10.3)	0.108
PAD (n, %)	62 (3.3)	19 (4.5)	0.236	99 (2.3)	18 (1.6)	0.110
COPD (n, %)	43 (2.3)	10 (2.4)	0.935	98 (2.3)	26 (2.3)	0.158
OMI (n, %)	348 (18.7)	101 (24.0)	0.013	749 (17.7)	211 (18.3)	0.619
Previous PCI (n, %)	480 (25.8)	133 (31.6)	0.015	976 (23.1)	247 (21.5)	0.250
Previous CABG (n, %)	82 (4.4)	21 (5.0)	0.602	163 (3.9)	25 (2.2)	0.006
Serum creatinine (ml/min)	74.9 ± 16.7	76.9 ± 17.8	0.233	75.4 ± 15.3	76.2 ± 15.5	0.425
HbA1c (%)	7.8 ± 1.4	7.9 ± 1.4	0.772	6.1 ± 0.6	6.1 ± 0.6	0.744
Clinical presentation (n, %)						
Stable angia	796 (42.7)	175 (41.6)	0.658	1631 (38.5)	432 (37.5)	0.533
NSTE-ACS	857 (46.0)	189 (44.9)	0.674	2026 (47.9)	534 (46.4)	0.373
STEMI	209 (11.2)	57 (13.5)	0.181	574 (13.6)	185 (16.1)	0.030
Medication (cases, %)						
Aspirin	1841 (98.9)	419 (99.5)	0.289	4173 (98.6)	1140 (99.0)	0.244
Clopidogrel	1840 (98.8)	416 (98.8)	> 0.999	4159 (98.3)	1138 (98.9)	0.153
DAPT	1821 (97.8)	415 (98.6)	0.311	4104 (97.0)	1131 (98.3)	0.018
GP IIIb/IIa inhibitors	206 (11.1)	62 (14.7)	0.035	500 (11.8)	217 (18.9)	< 0.001
Statin	1774 (95.3)	406 (96.4)	0.299	4071 (96.2)	1104 (95.9)	0.663
β‑blocker	1705 (91.6)	403 (95.7)	0.004	3782 (89.4)	1021 (88.7)	0.521
Calcium antagonist	956 (51.3)	221 (52.5)	0.669	2002 (47.3)	518 (45.0)	0.165
Nitrate	1807 (97.0)	414 (98.3)	0.141	4130 (97.6)	1127 (97.9)	0.518
PPI	341 (18.3)	79 (18.8)	0.829	836 (19.8)	266 (23.1)	0.012
Therapeutic status for DM (n, %)						
Diet and exercise	342 (18.4)	76 (18.1)	0.880	–	–	–
Antidiabetic agents	836 (44.9)	209 (49.6)	0.078	–	–	–
Insulin	494 (26.5)	104 (24.7)	0.441	–	–	–
Insulin and antidiabetic agents	190 (10.2)	32 (7.6)	0.104	–	–	–

*DM* diabetes mellitus, *BP-DES* biodegradable polymer drug-eluting stents, *G2-DES* second generation drug-eluting stents, *BMI* body mass index, *LVEF* left ventricular ejection fraction, *PAD* peripheral arterial disease, *COPD* chronic obstructive pulmonary disease, *OMI* old myocardial infarction, *PCI* percutaneous coronary intervention, *CABG* coronary artery bypass grafting, *NSTE-ACS* non-ST-segment elevation acute coronary syndrome, *STEMI* ST-segment elevation myocardial infarction, *DAPT* dual antiplatelet treatment, *GP* glycoprotein, *PPI* proton pump inhibitors

Angiographic and procedural characteristics were generally similar between patients with DM in the BP-DES and G2-DES groups (all P > 0.05). However, patients without DM in the BP-DES group had a significantly greater number of lesions treated (P = 0.013) and a higher prevalence of thrombolysis in myocardial infarction 0 flow before PCI (P < 0.001), B2 or C lesions (P = 0.013), and chronic total occlusion lesions (CTO) (P < 0.001) compared with the G2-DES group (Table 2).

**Table 2:** Angiographic and procedural characteristics

Characteristics	DM		*P*	No-DM		*P*
	G2‑DES N = 1862	BP‑DES N = 421		G2‑DES N = 4232	BP‑DES N = 1151	
Lesions involving LM	46 (2.5)	9 (2.1)	0.688	81 (1.9)	15 (1.3)	0.165
Lesions involving LAD	1703 (91.5)	377 (89.5)	0.213	3929 (92.8)	1050 (91.2)	0.065
Lesions involving LCX	308 (16.5)	56 (13.3)	0.101	565 (13.4)	163 (14.2)	0.476
Lesions involving RCA	373 (20.0)	94 (22.3)	0.292	713 (16.8)	183 (15.9)	0.444
Number of lesions treated	1.4 ± 0.6	1.4 ± 0.6	0.664	1.3 ± 0.59	1.34 ± 0.60	0.013
Number of stents	1.8 ± 1.0	1.7 ± 0.9	0.177	1.75 ± 0.89	1.67 ± 0.88	0.391
Number of target vessel						
Single vessel	386 (20.7)	70 (16.6)	0.057	1320 (31.2)	340 (29.5)	0.282
Double vessel	576 (30.9)	143 (34.0)	0.578	1316 (31.1)	379 (32.9)	0.236
Triple vessel	779 (41.8)	185 (43.9)	0.429	1349 (31.9)	378 (32.8)	0.534
SYNTEX score	11.7 ± 8.0	11.9 ± 8.2	0.684	10.9 ± 7.7	11.0 ± 7.8	0.329
Normal origin of coronary artery	1774 (95.3)	403 (95.7)	0.692	4051 (95.7)	1102 (95.7)	0.977
Right distribution of coronary artery	1633 (87.7)	382 (90.7)	0.081	3747 (88.5)	1019 (88.5)	0.994
Transradial approach	1664 (89.4)	375 (89.1)	0.861	3813 (90.1)	1011 (87.8)	0.026
Pulling out sheath directly	1408 (75.6)	328 (77.9)	0.320	3277 (77.4)	904 (78.5)	0.424
IVUS application	97 (5.2)	16 (3.8)	0.229	218 (5.2)	58 (5.0)	0.878
IABP application	25 (1.3)	5 (1.2)	0.801	40 (0.9)	12 (1.0)	0.765
TIMI flow before PCI						
0	321 (17.2)	71 (16.9)	0.995	678 (16.0)	235 (20.4)	< 0.001
1	61 (3.3)	20 (4.8)	0.626	123 (2.9)	35 (3.0)	0.811
2	221 (11.9)	36 (8.6)	0.290	483 (11.4)	125 (10.9)	0.599
3	1259 (67.6)	294 (69.8)	0.247	2948 (69.7)	756 (65.7)	0.010
TIMI flow after PCI						
0	21 (1.1)	6 (1.4)	0.593	41 (1.0)	12 (1.0)	0.822
1	3 (0.2)	0 (0)	> 0.999	9 (0.2)	3 (0.3)	0.728
2	10 (0.5)	4 (1.0)	0.226	30 (0.7)	11 (1.0)	0.393
3	1828 (98.2)	411 (97.6)	0.754	4152 (98.1)	1125 (97.7)	0.425
B2 or C lesions	1430 (76.8)	330 (78.4)	0.484	3101 (73.3)	885 (76.9)	0.013
severe calcification	72 (3.9)	13 (3.1)	0.446	115 (2.7)	29 (2.5)	0.712
CTO lesions	143 (7.7)	30 (7.1)	0.698	267 (6.3)	107 (9.3)	< 0.001
Ostial lesions	316 (17.0)	66 (15.7)	0.521	697 (16.5)	178 (15.5)	0.413
Bifurcation lesions	346 (18.6)	77 (18.3)	0.889	825 (19.5)	239 (20.8)	0.337
Thrombosis	67 (3.6)	15 (3.6)	0.972	173 (4.1)	50 (4.3)	0.699

*DM* diabetes mellitus, *BP*-*DES* biodegradable polymer drug-eluting stents, *G2*-*DES* second generation drug-eluting stents, *LM* left main, LAD left anterior descending, *LCX* left circumflex, *RCA* right coronary artery, *SYNTEX* synergy between percutaneous coronary interventions with TAXUS and cardiac surgery, *IVUS* intravascular ultrasound, *IABP* intra-aortic balloon pump, *TIMI* thrombolysis in myocardial infarction, *PCI* percutaneous coronary intervention, *CTO* chronic total occlusion

### Two-year clinical outcomes of G2-DESs versus BP-DESs

**Fig. 2:**
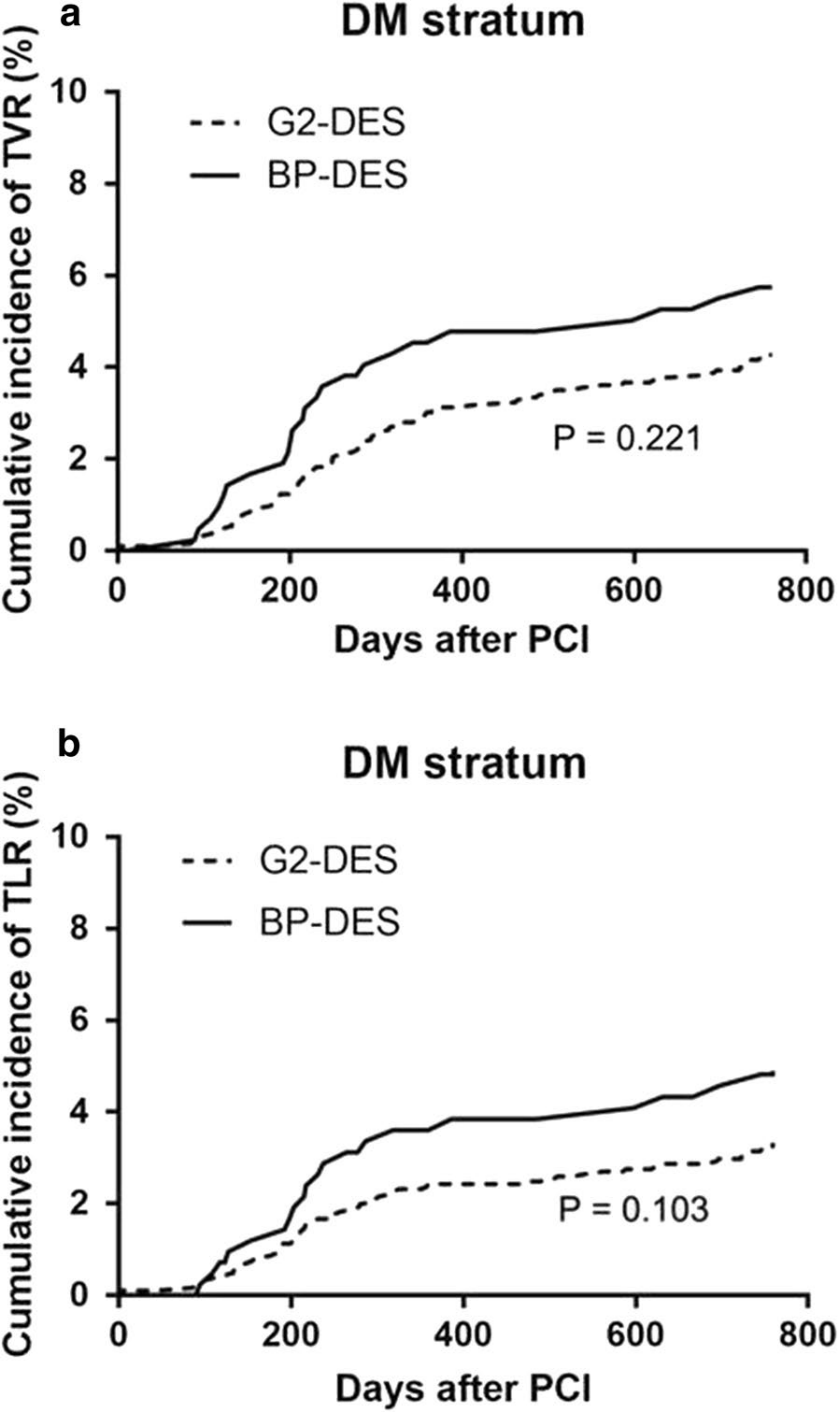
Kaplan–Meier survival curves in **a** TVR and **b** TLR in patients with DM. *TVR* target vessel revascularization, *TLR* target lesion revascularization, *DM* diabetes mellitus

**Fig. 3:**
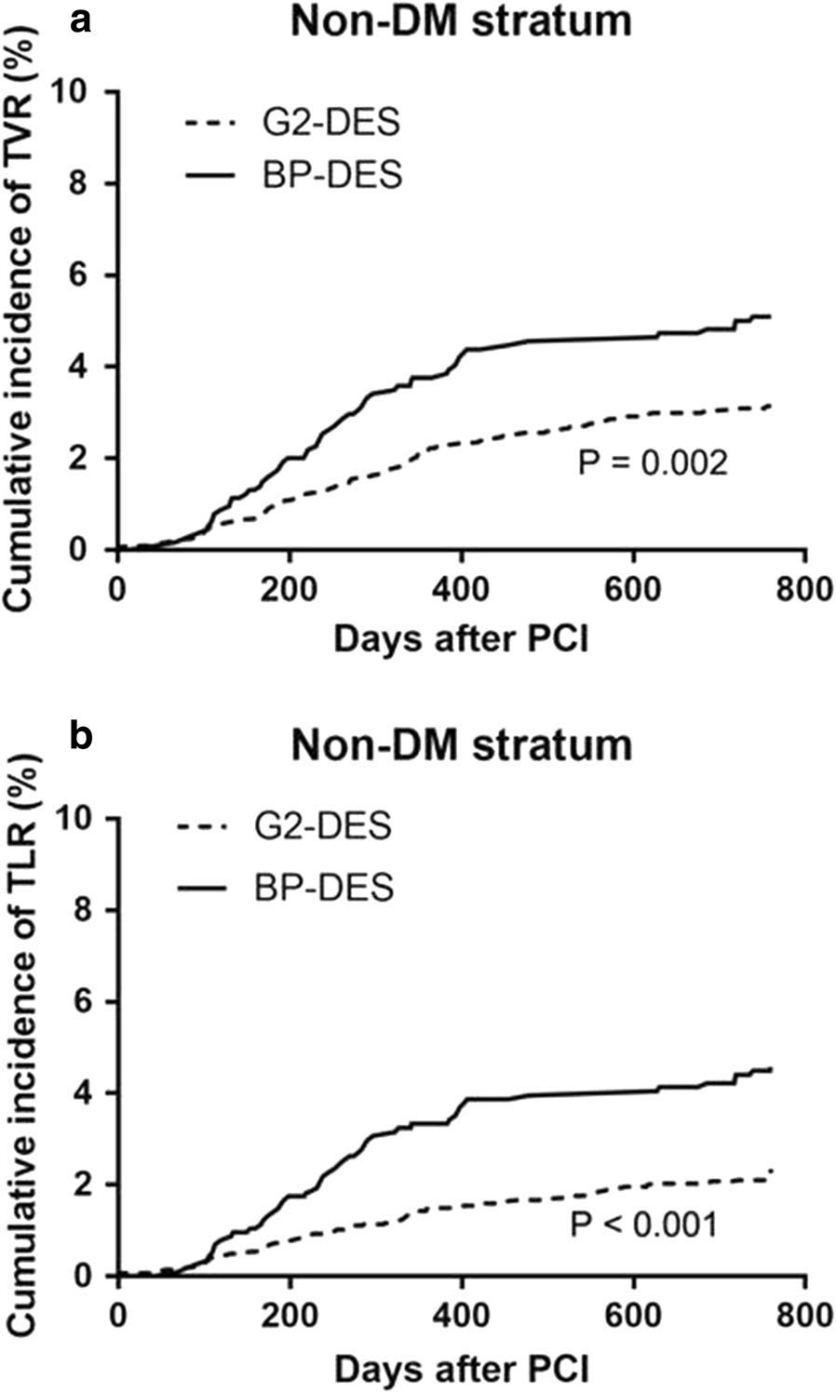
Kaplan–Meier survival curves in **a** TVR and **b** TLR in patients without DM. *TVR* target vessel revascularization, *TLR* target lesion revascularization, *DM* diabetes mellitus

**Fig. 4:**
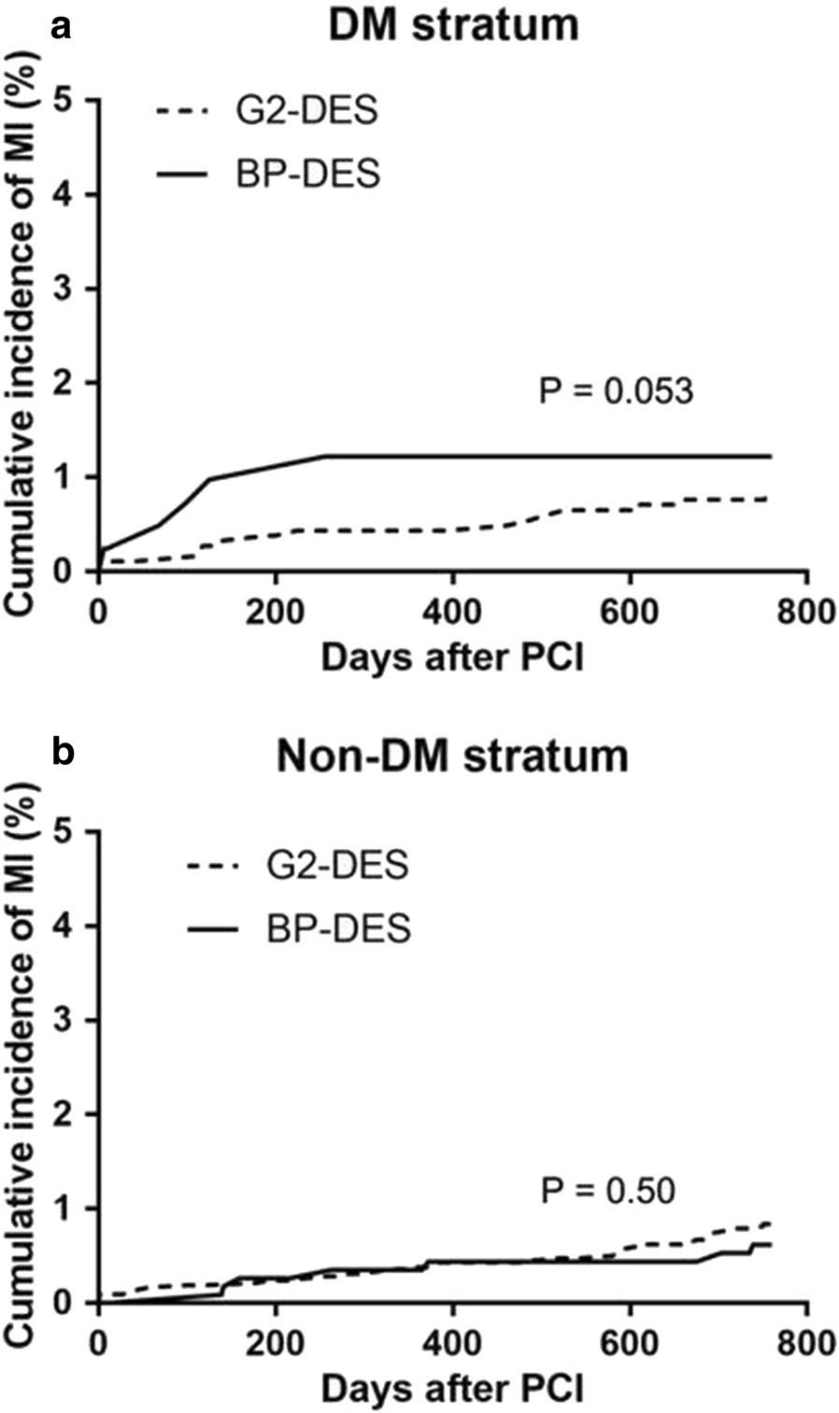
Kaplan–Meier survival curves in MI in **a** patients with DM and **b** without DM. *MI* myocardial infarction, *DM* diabetes mellitus

In patients with DM, the incidence of all-cause death, cardiac death, stent thrombosis, TVR, TLR, stroke, and bleeding were not significantly different between the BP-DES and G2-DES groups at the 2-year follow-up (P > 0.05). Furthermore, in patients with DM, the incidence of non-fatal MI had the higher tended in the G2-DES group compared with the BP-DES group (P = 0.05) (Table 3). In patients without DM, the incidence of all-cause death, cardiac death, non-fatal MI, stent thrombosis, stroke, and bleeding was not significantly different between the BP-DES and G2-DES groups (P > 0.05). However, the incidence of TVR (5.1% vs. 3.2%, P = 0.002) and TLR (4.5% vs. 2.2%, P < 0.001) was significantly higher in the BP-DES group than in the G2-DES group in the 2-year follow-up (Table 3). Kaplan–Meier analysis of the cumulative incidence of TVR and TLR showed no significant difference between the BP-DES and G2-DES groups in patients with DM (P = 0.221, P = 0.103, respectively) (Fig. 2). Kaplan–Meier analysis showed that the cumulative incidence of TVR and TLR was significantly greater in the BP-DES group than in the G2-DES group in patients without DM (P = 0.002, P < 0.001, respectively) (Fig. 3). Regardless of whether patients had DM, Kaplan–Meier analysis of the cumulative incidence of non-fatal MI showed no significant difference between the BP-DES and G2-DES groups (Fig. 4).

**Table 3:** Follow-up on patients at 2 years clinical outcomes

Clinical outcomes (n, %)	DM			No-DM		
	G2‑DES N = 1862	BP‑DES N = 421	*p*	G2‑DES N = 4232	BP‑DES N = 1151	*P*
MACE	219 (11.8)	60 (14.3)	0.159	403 (9.5)	129 (11.2)	0.089
All-cause death	27 (1.5)	6 (1.4)	0.969	40 (0.9)	16 (1.4)	0.187
Cardiac death	16 (0.9)	2 (0.5)	0.554	25 (0.6)	6 (0.5)	0.782
Non-fatal MI	37 (2.0)	15 (3.6)	0.050	75 (1.8)	17 (1.5)	0.493
ST	20 (1.1)	5 (1.2)	0.797	32 (0.8)	6 (0.5)	0.399
Revascularization	145 (7.8)	39 (9.3)	0.315	279 (6.6)	89 (7.7)	0.174
TVR	81 (4.4)	24 (5.7)	0.232	136 (3.2)	59 (5.1)	0.002
TLR	66 (3.5)	22 (5.2)	0.106	92 (2.2)	52 (4.5)	< 0.001
Stroke	26 (1.4)	4 (1.0)	0.468	51 (1.2)	15 (1.3)	0.789
Bleeding	124 (6.7)	21 (5.0)	0.204	310 (7.3)	79 (6.9)	0.592
BARC 2_5	50 (2.7)	8 (1.9)	0.355	123 (2.9)	31 (2.7)	0.701
BARC 3_5	8 (0.4)	1 (0.2)	> 0.999	24 (0.6)	4 (0.3)	0.358

*DM* diabetes mellitus, *G2*-*DES* second generation drug-eluting stent, *BP*-*DES* biodegradable polymer drug-eluting stent, *MACE* major adverse cardiac events, *MI* myocardial infarction, *ST* stent thrombosis, *TVR* target vessel revascularization, *TLR* target lesion revascularization, *BARC* Bleeding Academic Research Consortium definition

In unadjusted univariate analysis, the 2-year cumulative incidence of all-cause death, cardiac death, non-fatal MI, stent thrombosis, TVR, TLR, stroke, and bleeding was not significantly different between the BP-DES and G2-DES groups in patients with DM (Table 4). In unadjusted univariate analysis, the cumulative incidence of all-cause death, cardiac death, stent thrombosis, stroke, and bleeding were not significantly different between the BP-DES and G2-DES groups in patients without DM. However, TVR and TLR were significantly higher in the BP-DES group than in the G2-DES group in patients without DM [hazard ratio (HR) 1.619, 95% confidence interval [CI] 1.193–2.198, P = 0.002; HR 2.109, 95% CI 1.501–2.963, P < 0.001, respectively] (Table 5).

**Table 4:** Univariate and multivariate Cox proportional hazard regression analysis for DM patients

Clinical outcomes (n, %)	Univariate multivariate			
	HR (95% CI)	*P*	HR (95% CI)	*P*
MACE	1.245 (0.935–1.656)	0.133	1.252 (0.939–1.669)	0.126
All-cause death	0.986 (0.407–2.389)	0.976	0.953 (0.387–2.351)	0.917
Cardiac death	0.555 (0.128–2.412)	0.432	0.410 (0.089–1.879)	0.251
Non-fatal MI	1.808 (0.992–3.295)	0.053	1.689 (0.913–3.122)	0.095
Stent thrombosis	1.116 (0.419–2.974)	0.826	1.002 (0.370–2.714)	0.997
Revascularization	1.205 (0.846–1.717)	0.300	1.215 (0.852–1.733)	0.283
TVR	1.329 (0.843–2.096)	0.221	1.325 (0.835–2.101)	0.232
TLR	1.495 (0.923–2.422)	0.103	1.453 (0.891–2.371)	0.134
Stroke	0.681 (0.237–1.950)	0.474	0.731 (0.253–2.111)	0.563
Bleeding	0.748 (0.471–1.188)	0.219	0.727 (0.447–1.182)	0.198
BRAC 2_5	0.710 (0.337–1.497)	0.368	0.705 (0.333–1.491)	0.360
BRAC 3_5	0.554 (0.069–4.432)	0.578	0.563 (0.070–4.535)	0.590

*DM* diabetes mellitus, *G2*-*DES* second generation drug-eluting stent, *BP*-*DES* biodegradable polymer drug-eluting stent, *MACE* major adverse cardiac events, *MI* myocardial infarction, *TVR* target vessel revascularization, *BARC* Bleeding Academic Research Consortium definition

**Table 5:** Univariate and multivariate Cox proportional hazard regression analysis for No-DM patients

Clinical outcomes (n, %)	Univariate multivariate			
	HR (95% CI)	*P*	HR (95% CI)	*P*
MACE	1.190 (0.976–1.451)	0.085	1.145 (0.937–1.401)	0.186
All-cause death	1.477 (0.827–2.637)	0.188	1.268 (0.686–2.344)	0.449
Cardiac death	0.886 (0.363–2.159)	0.790	0.772 (0.293–2.039)	0.602
Non-fatal MI	0.834 (0.493–1.413)	0.50	0.758 (0.441–1.304)	0.317
Stent thrombosis	0.692 (0.289–1.654)	0.407	0.573 (0.222–1.480)	0.250
Revascularization	1.188 (0.936–1.508)	0.157	1.184 (0.931–1.505)	0.168
TVR	1.619 (1.193–2.198)	0.002	1.539 (1.128–2.099)	0.007
TLR	2.109 (1.501–2.963)	< 0.001	1.963 (1.390–2.772)	< 0.001
Stroke	1.087 (0.611–1.932)	0.778	0.976 (0.538–1.771)	0.936
Bleeding	1.022 (0.798–1.310)	0.861	1.055 (0.807–1.379)	0.696
BRAC 2_5	0.927 (0.625–1.374)	0.705	0.929 (0.625–1.381)	0.714
BRAC 3_5	0.613 (0.213–1.766)	0.364	0.630 (0.216–1.839)	0.397

*DM* diabetes mellitus, *G2*-*DES* second generation drug-eluting stent, *BP*-*DES* biodegradable polymer drug-eluting stent, *MACE* major adverse cardiac events, *MI* myocardial infarction, *TVR* target vessel revascularization, *BARC* Bleeding Academic Research Consortium definition

After multivariable analysis with adjustment for age, sex, body mass index, left ventricular ejection fraction, serum creatinine levels, use of a proton pump inhibitor and GPIIa/IIIb, SYNTEX score, a history of coronary heart disease, previous PCI and CABG, OMI, hypertension, current smoker, multivessel disease, B2 or C lesions, the number of target lesions, the transradial approach, and CTO, use of a BP-DES was still not an independent predictor of TLR in patients with DM (HR 1.453, 95% CI 0.891–2.371, P = 0.134) (Table 4). For clinical endpoints that were measured, current smoker, a history of coronary heart disease, and old myocardial infarction were independent predictors of TLR in patients with DM (Additional file 1: Fig. S1). However, before and after applying multivariable analysis, use of a BP-DES was still an independent predictor of TLR in patients without DM (HR 1.963, 95% CI 1.390–2.772, P < 0.001) (Table 5). Furthermore, B2 or C lesions and the SYNTAX score were independent predictors of TLR in patients without DM (Additional file 2: Fig. S2).

## Discussion

### Main findings in this study

Our prospective, observational study from a high-volume cardiovascular center in China compared the efficacy and safety between G2-DESs and BP-DESs in a large group of patients with and without DM. Based on a 2-year follow-up, our major findings were as follows: (1) G2-DESs were associated with a lower risk of TLR, which suggested better efficacy, and G2-DESs had a similar safety profile to that of BP-DESs in patients without DM; and (2) among patients with DM, G2-DESs had similar efficacy and safety profiles to those of BP-DESs.

### Relationship between several types of DESs and adverse cardiac events

G2-DESs are characterized by novel stent platforms, more lipophilic sirolimus analogues, and/or more biocompatible durable polymers. These advantages of durable polymer G2-DESs enabled addition. The design of the stent platform may also shed light on differences in efficacy. The rate of TLR in patients with lesion calcification was lower with G2-DESs than with G1-DESs [21]. Additionally, lower strut thickness was associated with improved re-endothelialization and arterial healing, [22] and arterial repair after G2-DES implantation into vulnerable plaques remains vulnerable, even at 1-year follow-up [23]. Biodegradable polymers, in contrast to durable polymers, can be completely metabolized, potentially reducing the risk of late complications, such as stent uncovering, malapposition, and endothelial dysfunction [24]. Some clinical trials have shown that BP-DESs have a low rate of TVR and TLR in real-world performance, [25, 26] even in high-risk patients, such as those with DM, small vessels, CTO, and AMI [27–29]. BP-DESs and G2-DESs could be effective alternatives for drug release from the metallic stent platform. Longer term follow-up might be required to demonstrate the potential benefits of BP-DESs relative to G2-DESs. Some large head-to-head trials and network meta-analyses have shown that safety and efficacy outcomes of BP-BESs were non-inferior to those of G2-DESs 1, 3, and 5 years after stent implantation [30–34].

### Efficacy and safety of several types of DESs in patients with and without DM

DM is still one of the major risk factors for stent restenosis [16]. Some studies have shown that patients with DM using BP-DESs, particularly those with insulin-dependent DM, have worse outcomes, such as TLR and mortality, than patients without DM [35, 36]. However, a unified view of the long-term clinical efficacy and safety of different generations of DESs among DM patients has not been well established. Long-term follow-up for at least 1 year is necessary to verify the efficacy and safety of DESs for patients with DM. Our study expands current knowledge of long-term clinical outcomes up to 2 years between G2-DES and BP-DES implantation according to the presence or absence of DM.

In our study, before and after multivariable adjustment, there were no differences in 2-year MACE between G2-DES and BP-DES implantation in patients with or without DM. Before and after multivariable adjustment, BP-DES implantation had almost a 1.5–2.0-fold higher risk of 2-year TLR compared with G2-DESs in patients without DM. However, there were no differences in 2-year TLR between G2-DES and BP-DES implantation in patients with DM, even after multivariable adjustment.

Some previous studies showed that G2-DESs had a lower risk for TLR compared with G1-DESs in patients with DM [37, 38]. This finding was not observed in BP-DES compared with G2-DES implantation in patients with DM in our study, [12, 39–41] G2-DESs had a lower risk for TLR compared with BP-DESs in patients without DM in our study. In patients with DM in our study, clinical conditions were more complex in BP-DES implantation, with a higher proportion of hypertension, OMI, and previous PCI than with G2-DES implantation. Additionally, in patients with DM, current smoker, a history of coronary heart disease, and OMI were independent predictors of TLR. Some study had shown that fluctuation of glucose levels may affect vessel healing after DES implantation in patients with CHD [42]. Therefore, fluctuations in glucose levels may be an important target for secondary prevention after coronary stenting. In our study, the average value of HbA1c was 7.8% and control of HbA1c was not ideal in patients with DM. Hence, further control was required. In patients without DM in our study, lesions were more complex in BP-DES implantation, with a higher proportion of CTO and B2 or C lesions. Additionally, B2 or C lesions and the SYNTAX score were independent predictors of TLR in patients without DM. The complexity of the lesion level may have affected the proportion of TLR after BP-DES implantation in patients without DM in our study.

In the current study, the strut thickness of the G2-DES was 81–91 μm. In contrast, most BP-DESs had a higher strut thickness of 120 μm (Excel) and 100 μm (BuMA), [43] which accounted for 79.9% of all BP-DESs used in the study. This may in part explain the finding in the current study that G2-DESs had a similar incidence of stent thrombosis as BP-DESs in patients with or without DM within 2 years after PCI. Additionally, patients in our study had good adherence to dual antiplatelet therapy after PCI. Up to 97.4% of patients received dual antiplatelet therapy for 1 year, which may have played an important role in preventing stent thrombosis and cardiogenic death.

### Limitations

There are several limitations in our single-center study. First, as in any non-randomized study, our research was limited by an imbalance in patients and selection of procedures. However, multivariate Cox regression was applied to minimize the dissymmetry between the groups. Second, all of the BP-DESs in our study were sirolimus-eluting stents because of their availability in our center, which limited the generalization of the conclusion to all BP-DES. Third, the fact that our study was from a single center hindered the statistical power. Additionally, a safety issue associated with durable polymers is typically represented by stent thrombosis. However, the 2-year follow-up in our study may not have fully addressed safety concerns in current practice. Therefore, we intended to perform a longer follow-up and update this investigation in the future.

## Conclusions

Our prospective, observational study shows that G2-DESs reduce the risk of TLR, and have a similar incidence of death and MI compared with BP-DESs in patients without DM at 2-year follow-up. G2-DESs are similar to BP-DESs regarding the incidence of death, TLR, and stent thrombosis in patients with DM. These findings suggest a similar efficacy and safety between these two types of stents.

## Additional files

**Additional file 1: Figure S1.** Multivariate analysis for TLR in patients with DM. TLR: target lesion revascularization, DM: diabetes mellitus, CHD: coronary heart disease, CTO: chronic total occlusion, OMI: old myocardial infarction, CABG: coronary artery bypass grafting, PCI: percutaneous coronary interventions, GP: glycoprotein, PPI: proton pump inhibitors, EF: ejection fraction, BMI: body mass index.

**Additional file 2: Figure S2.** Multivariate analysis for TLR in patients without DM. TLR: target lesion revascularization, DM: diabetes mellitus, SYNTEX: Synergy between percutaneous coronary interventions with TAXUS and Cardiac Surgery, CHD: coronary heart disease; CTO: chronic total occlusion; OMI: old myocardial infarction; CABG: coronary artery bypass grafting; PCI: PCI: percutaneous coronary interventions; GP: glycoprotein; PPI: proton pump inhibitors; EF: ejection fraction; BMI: body mass index.

## Abbreviations

BARC: Bleeding Academic Research Consortium
BMS: bare metal stents
BP-DES: biodegradable polymer drug eluting stents
CABG: coronary artery bypass grafting
CTO: chronic total occlusion
DM: diabetes mellitus
MACE: major adverse cardiac events
MI: myocardial infarction
OMI: old myocardial infarction
PCI: percutaneous coronary interventions
G2-DES: second-generation drug eluting stents
SYNTEX: synergy between percutaneous coronary interventions with TAXUS and cardiac surgery
TLR: target lesion revascularization
TVR: target vessel revascularization
