# Supplementary figures and images for: Biodegradable polymer drug-eluting stents versus second-generation drug-eluting stents in patients with and without diabetes mellitus: a single-center study
Source: Cardiovasc Diabetol. 2018 Aug 14;17:114. doi: 10.1186/s12933-018-0758-0 (PMC6090623; doi:10.1186/s12933-018-0758-0)

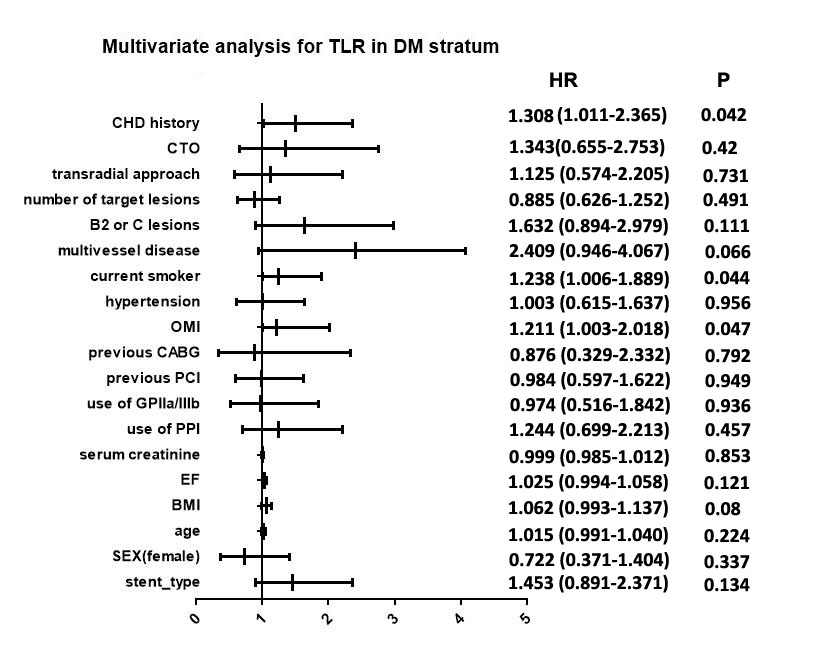

Supplement: Supplementary file 1 — Additional file 1: Figure S1. Multivariate analysis for TLR in patients with DM. TLR: target lesion revascularization, DM: diabetes mellitus, CHD: coronary heart disease, CTO: chronic total occlusion, OMI: old myocardial infarction, CABG: coronary artery bypass grafting, PCI: percutaneous coronary interventions, GP: glycoprotein, PPI: proton pump inhibitors, EF: ejection fraction, BMI: body mass index. [file 12933_2018_758_MOESM1_ESM.jpg]

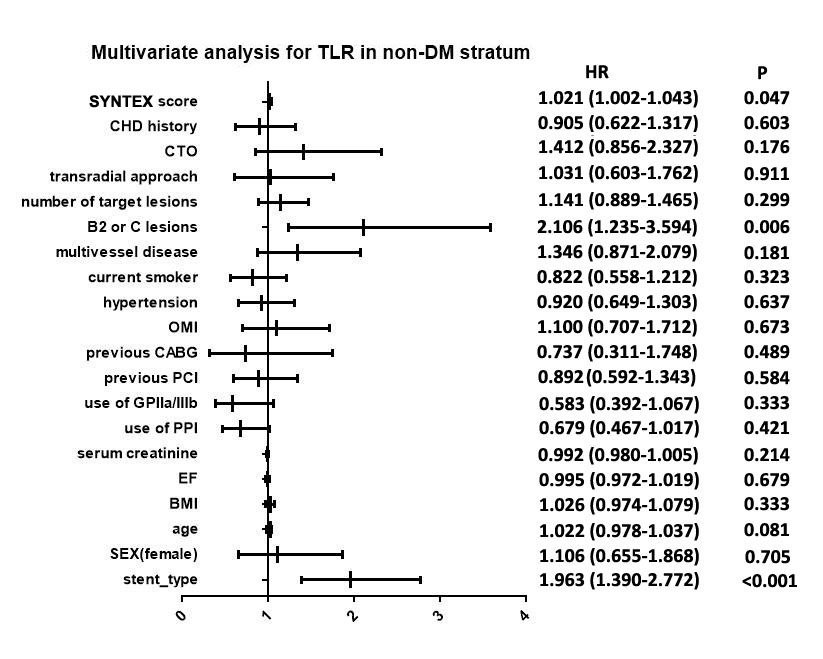

Supplement: Supplementary file 2 — Additional file 2: Figure S2. Multivariate analysis for TLR in patients without DM. TLR: target lesion revascularization, DM: diabetes mellitus, SYNTEX: Synergy between percutaneous coronary interventions with TAXUS and Cardiac Surgery, CHD: coronary heart disease; CTO: chronic total occlusion; OMI: old myocardial infarction; CABG: coronary artery bypass grafting; PCI: PCI: percutaneous coronary interventions; GP: glycoprotein; PPI: proton pump inhibitors; EF: ejection fraction; BMI: body mass index. [file 12933_2018_758_MOESM2_ESM.jpg]
